# Effect of both the 9S management theory and the hierarchical management model on management of intensive care unit

**DOI:** 10.1590/1980-220X-REEUSP-2024-0114en

**Published:** 2025-01-13

**Authors:** Mengmeng Pan, Xiaoying Zeng, Huirong Liu, Xiaomin Zheng

**Affiliations:** 1 The First Affiliated Hospital of Wenzhou Medical University, Intensive Care Unit, Wenzhou, China.; 2 The First Affiliated Hospital of Wenzhou Medical University, Wenzhou, China.; 3 The First Affiliated Hospital of Wenzhou Medical University, Department of Critical Care Medicine, Wenzhou, China.

**Keywords:** Patient Comfort, Intensive Care Units, Organization and Administration, Nursing, Quality of Health Care, Conforto do Paciente, Unidades de Terapia Intensiva, Organização e Administração, Enfermagem, Qualidade da Assistência à Saúde, Comodidad del Paciente, Unidades de Cuidados Intensivos, Organización y Administración, Enfermería, Calidad de la Atención de Salud

## Abstract

**Objective:**

To explore the effect of the 9S management theory plus hierarchical management model on the management of intensive care unit (ICU).

**Method:**

Ninety patients hospitalized in ICU from January 2021 to January 2023 were selected and divided into a control group (n = 45) and a study group (n = 45) according to different ward management methods. The management lasted for one month. The ward management quality score, nursing quality score, level of comfort, severity of disease, and incidence of adverse events were compared between the two groups.

**Results:**

The study group had higher ward management quality scores of all items than those of the control group after management (P < 0.05). In the study group, the nursing quality scores of all items and General Comfort Questionnaire cores were higher, while the acute physiology and chronic health evaluation II scores were lower than those in the control group after management (P < 0.05). The incidence rate of adverse reactions during management in the study group was not significantly different from that of the control group (P > 0.05).

**Conclusion:**

Both the 9S management theory and hierarchical management model can effectively improve the ward management quality and nursing quality in ICU management.

## RESUMO

**Objetivo:**

Explorar o efeito da teoria de gestão 9S juntamente com o modelo de gestão hierárquica na gestão da unidade de terapia intensiva (UTI).

**Método:**

Noventa pacientes hospitalizados na UTI de janeiro de 2021 a janeiro de 2023 foram selecionados e divididos em um grupo controle (n = 45) e um grupo de estudo (n = 45) de acordo com diferentes métodos de gestão de enfermaria. A gestão durou um mês. O escore de qualidade da gestão da enfermaria, o escore de qualidade da enfermagem, o nível de conforto, a gravidade da doença e a incidência de eventos adversos foram comparados entre os dois grupos.

**Resultados:**

O grupo de estudo teve escores de qualidade da gestão da enfermaria mais altos de todos os itens do que aqueles do grupo controle após a gestão (P < 0,05). No grupo de estudo, os escores de qualidade da enfermagem de todos os itens e os núcleos do Questionário de Conforto Geral foram maiores, enquanto os escores de fisiologia aguda e avaliação crônica da saúde II foram menores do que aqueles no grupo controle após a gestão (P < 0,05). A taxa de incidência de reações adversas durante a gestão no grupo de estudo não foi significativamente diferente daquela do grupo controle (P > 0,05).

**Conclusão:**

A teoria de gestão 9S juntamente ao modelo de gestão hierárquica podem efetivamente melhorar a qualidade da gestão da enfermaria e a qualidade da enfermagem na gestão da UTI.

## RESUMEN

**Objetivo:**

Explorar el efecto de la teoría de gestión 9S en conjunto con el modelo de gestión jerárquica en la gestión de la unidad de cuidados intensivos (UCI).

**Método:**

Se seleccionaron noventa pacientes hospitalizados en la UCI entre enero de 2021 y enero de 2023 y se los dividió en dos grupos, control (n = 45) y estudio (n = 45), según diferentes métodos de gestión de la sala de enfermería. La gestión duró un mes. Se compararon la puntuación de calidad de la gestión de la sala, la puntuación de calidad de la labor de enfermería, el nivel de confort, la gravedad de la enfermedad y la incidencia de acontecimientos adversos entre los dos grupos.

**Resultados:**

En el grupo de estudio, las puntuaciones de calidad de enfermería de todos los ítems y de los núcleos del Cuestionario de Confort General fueron altas y las puntuaciones de fisiología aguda y de evaluación de la salud crónica II fueron inferiores a las del grupo de control tras la gestión (P < 0,05). La tasa de incidencia de reacciones adversas durante la gestión en el grupo de estudio no fue significativamente diferente de la del grupo de control (P > 0,05).

**Conclusión:**

La teoría de gestión 9S en conjunto con el modelo de gestión jerárquica pueden mejorar eficazmente la calidad de la gestión de las salas y de la enfermería en la gestión de la UCI.

## INTRODUCTION

The intensive care unit (ICU) is a comprehensive department dedicated to treating patients with acute reversible diseases, high-risk factors, acute exacerbation of chronic diseases, and irreversible deterioration of acute and chronic diseases^(1)^. Due to its fast pace, high intensity and high-risk nature, ward management in ICU faces significant challenges. In recent years, patients and their families have developed a more sophisticated understanding of medical care, leading to higher nursing and management expectations for medical staff and wards^(2,3)^. To fully meet the patients’ evolving needs of nursing and management and to enhance competitiveness, the ward management model is continuously evolving. Therefore, higher management standards are required for medical staff. They should not only strengthen their professional nursing skills but also enhance comprehensive management of the ward environment, facilities, equipment, and consumables.

Currently, in order to ensure the normal and orderly operation of the ward, routine programmed management people are employed in ICU. However, the optimization of human, financial and material resources in the management process have not yet been achieved, indicating the need for a more efficient management model. The 9S management concept, developed from the 5S management concept, includes SEIRI, SEITON, SEISO, SEIKETSU, SAVING, SAFETY, SERVICE, SATISFACTION and SHITSUKE^(4)^. The 9S management theory introduces a modern centralized concept that can effectively improve operational efficiency and control resource loss, leading to better management outcomes^(5)^. Applying the 9S management concept in enterprise management can strengthen employees’ individual quality, enhance group harmony, and effectively improve overall satisfaction^(6)^. As the medical service industry evolves, the 9S management concept has gradually been integrated into all processes of hospital management, demonstrating its great significance as a guiding model, besides being of practical value^(7)^.

Additionally, the hierarchical management model, with its authorization and auditing system based on management classes, effectively clarifies management responsibilities and enhances management efficiency^(8,9)^. This model is also frequently used in hospitals, implementing targeted management measures for patients based on the severity of their conditions, which positively impacts management quality^(10,11)^. Until now, the 9S management theory has never been combined with hierarchical management model for ICU management.

Thereby motivated, we herein assessed the impact of combining the 9S management theory with hierarchical management model on ICU management, aiming to further investigate the cumulative effects and potential improvements in management outcomes.

## METHOD

### Subjects

In this retrospective study, 90 patients who had been hospitalized in the ICU of our hospital, from January 2021 to January 2023, were selected and divided into a control group (n = 45) and a study group (n = 45) according to different ward management methods. The sample size was determined according to the equation n = 2×(Z_α/2_ + Z_β_)^2^×σ^2^/∆^2^: where n is the sample size per group, Z_α/2_ is the critical value of the standard normal distribution at the chosen significance level, Z_β_ is the critical value of the standard normal distribution for the chosen power, σ is the standard deviation of the outcome variable, and Δ is the minimum detectable difference between the two groups.

### Inclusion and Exclusion Criteria

Inclusion criteria were as follows: 1) patients who met the criteria for referral to the ICU by clinical diagnosis, 2) those with complete clinical data, 3) those with normal cognitive function, communication ability and visual and auditory senses, 4) those aged 20–70 years old, and 5) those who and/or whose family members were informed about the study and voluntarily signed the informed consent form.

Exclusion criteria included: 1) patients with neurologic or psychiatric disorders, 2) those in coma, 3) those with severe contagious infectious diseases, or 4) those who withdrew from the study midway.

### Management Method for Control Group

The routine programmed management employed was: (1) Diagnosis and treatment management: a) After the patients were admitted to ICU, the responsible physicians and expert physicians were responsible for the treatment management, and the head nurse and the responsible nurses were responsible for the nursing management. The management personnel jointly discussed the treatment suggestions and cooperated after the joint ward rounds every day. b) The patients who needed consultations should be reported to the Medical Department by the expert physician in accordance with the procedures, and then the consultations were organized by the Medical Department. The responsible physician should organize the consultations for the patients who needed rescue. When the patient’s condition became stable, he/she should be promptly transferred back to the original department for treatment.

(2) Visit management: a) All personnel entering the ward must strictly adhere to aseptic operation and disinfection procedures. b) Ward rounds and handling of medical orders were performed before 10:00 in the morning, so no visitors and guests were allowed. c) The meal delivery and visiting time were specified. It was prohibited to enter the ward at non-specified time, and only one family member was allowed to enter the ward each time. The visiting and accompanying system should be strictly complied with after entering the ward, such as not walking around, and maintaining a clean and quiet room. d) A critically ill notice was required for visiting, and the physician was responsible for scheduling the visit.

(3) Daily nursing management: a) The responsible nurses needed to understand the conditions and diagnosis and treatment status of patients under their charge, cooperate with physicians to complete treatment and nursing care, closely observe the condition changes of patients, especially those who needed special monitoring, timely report and carry out necessary treatment in critical situations. b) The nurses needed to complete the work following the doctor’s orders, make records in a serious manner of special nursing, strictly implement the shift turnover and disinfection and isolation systems, and comply with the operating procedures. c) The nurses needed to check at any time, prepare all drugs and equipment required for first aid, and rapidly and accurately cooperate with the doctor to carry out rescue. d) The nurses needed to check at any time to ensure that the pipeline was unobstructed, ensure that the intravenous infusion rate was reasonable, and accurately calculate the dosage of special drugs, which should be verified by another personnel. The catheter should be well placed to avoid accidental extubation or falling and kept open for the patients who needed urethral catheter placement. The hourly urine volume and 24h intake and output volume were recorded, and routine nursing was given. e) The bed sheets were changed daily to keep beds and wards neat and clean, and the medical equipment was cleaned and sterilized regularly. f) Shift turnover should be performed seriously and accurately. In addition to writing a shift report, bedside-shit must be done.

### Management Method for Study Group

Both the 9S management theory and the hierarchical management model were employed. 9S management theory: (1) SEIRI and SEITON: a) A 9S management team was established, directed by the head nurse, and consisting of responsible nurses, expert physicians, and responsible physicians. b) The team members rationally planned the functional zoning of the ICU, and counted and recorded the quantity, performance and validity date of all items, equipment and consumables in the ward. According to the functional attributes, the items and equipment were reasonably placed in a fixed position, and the labels (name, number) were pasted. After use, the items and equipment were placed back according to the labels promptly. Medical consumables were distinguished and labelled according to the degree of risk, and they should be checked regularly for usage and supplemented. c) A fixed number of spare medicines were managed, the principle of “first-in-first-out” was followed, and regular verification was needed to ensure that the medicines were not packaged in a nude and mixed way, and they were not expired or deteriorated. High-risk medicines, confusing medicines and dangerous goods were placed with warning signs, and first-aid drugs were specially placed and managed by specialized personnel.

(2) SEISO and SEIKETSU: a) The cleaning staff was responsible for the hygiene of the ward and wiping all the items and facilities in the ward, once a day. b) The cleaning day was on Fridays, every week, and the head nurse was responsible for arrangement of equipment to clean up the hygienic dead angle. The nurse on duty assisted the cleaning staff in managing the ward environment at other times. The medical staff in the ward should not do anything that could jeopardize the cleaning.

(3) SAVING: Medical consumables should be placed in order according to their validity date, accounts should be established in the ward storehouse and dressing room, and accounts should be kept promptly after receiving medical consumables. The responsible physician should apply for next week’s consumables demands according to the number of patients and the frequency of treatments, avoiding expiration and waste.

(4) SAFETY: a) Disinfection and isolation system should be strictly implemented, and air, floor and table should be regularly disinfected (once every 2 hours) to ensure that the total bacterial count in the indoor air < 200/m^3^. b) Three districts were divided, the channels for personnel and material flows were rationally set up, and the ward environment should reach the class 2 standard (temperature: 20–22°C, humidity: 50–60%). c) Instruments and equipment should be regularly maintained by specialized personnel to ensure normal operation. The garbage and medical waste were handled strictly according to the requirements. The environment and facilities in the ward should be regularly inspected by specialized personnel to eliminate potential safety hazards.

(5) SHITSUKE: The key staff of the ward was regularly sent to “AAA” hospitals in and out of the province to study, shared their experiences after learning, and organized video-watching for nurses. Special learning activities of medical technology and medical ethics were regularly carried out, and theoretical (additional assessment of the nursing staff’s sense of responsibility and morality, sense of pride, etc.) and practical assessments were regularly held. The nurses who obtained excellent results were rewarded, or they would be punished and receive individual counselling and make-up examinations until obtaining qualified results.

(6) SERVICE: Hierarchical training was regularly given to the nursing staff to improve their nursing skills. A comprehensive ward-round system was implemented, and follow-up services were provided by telephone and WeChat after the patient was transferred out of the ward, so as to provide high-quality nursing service.

(7) SATISFACTION: A questionnaire survey was conducted on patients transferred out of the ward to understand the reasons for patient dissatisfaction, improve the process, and increase nursing satisfaction. Hierarchical management model: According to the critical conditions of patients, they were divided into different grades and labelled accordingly: Grade I (very critical), Grade I (more severe) and Grade III (moderately severe), and different management measures were taken for different grades of patients. Grade I management: The patients were not allowed to be visited but treated and cared for by specialized nurses and physicians. 24-h monitoring was required and their conditions were recorded on the special nursing sheet. The problems in the patient’s treatment and nursing were fully assessed, and preventive measures were taken in advance to prevent complications. Grade II management: The patients were allowed to be visited, the nurses and physicians were responsible for treatment and nursing, respectively, and the nursing staff completed the work following the doctor’s orders in a reasonable and orderly manner and regularly monitored and recorded the patients’ vital signs. Grade III management: Physicians made daily ward rounds to learn about the patient’s condition and give medical advice, and nursing staff routinely carried out daily nursing. The patient was transferred out of ICU when meeting the indication for transferring out. The management lasted for one month in both groups.

### Ward Management Quality Score

The hospital’s self-developed ward management quality scale was used to assess the management quality, which consisted of four items [hygienic environment, management of items (instruments, equipment, and consumables), treatment and nursing level of medical staff, and implementation of safety measures], with 25 points for each item. A higher score suggested a better management quality. The scale was validated by experts which yielded a validity index of 0.83 and a reliability index of 0.86.

### Nursing Quality Score

The hospital’s self-developed nursing quality scale was used to assess the nursing quality, including service attitude, theoretical assessment, practical assessment and nursing satisfaction, with 25 points for each item. A higher score meant a better nursing quality. The scale was validated by experts which yielded a validity index of 0.85 and a reliability index of 0.82.

### Comfort Assessment

The Simplified General Comfort Questionnaire (GCQ) developed by Kolcaba was used to assess patient comfort before and after management^(12)^, with a total of 28 items in four dimensions (physiology, psychology, environment and social culture). Using a 4-point scale, a higher score suggested better comfort.

### Assessment of Disease Severity

The severity of disease before and after management was assessed using the acute physiology and chronic health evaluation II (APACHE II) score^(13)^. APACHE II included acute physiology score, age score, and chronic health score, with 0–71 points, and a higher score suggested higher severity.

### Observation of Adverse Events

Delayed or incorrect implementation of medical orders, cross-infection, unplanned extubation, insufficient equipment power, and insufficient consumables were recorded.

### Statistical Analysis

SPSS22.0 software was used for statistical analysis. Measurement data were described by mean ± standard deviation ( $\bar{X}$ ± s) and subjected to the *t*-test. Count data were described by percentage and subjected to the χ^2^ test. P < 0.05 was considered statistically significant.

### Ethical Aspects Subsection

This study has received ethical approval from the hospital, and all patients signed an informed consent form (approval No. ZJWZFA20213402).

## RESULTS

### GENERAL DATA

The general data such as gender, age, duration of ICU stay, and APACHE II score were comparable between the two groups (P > 0.05) (Table 1).

**Table 1:** Baseline data of two groups – Wenzhou, Zhejiang Province, China, 2021–2023.

Baseline data		Control group (n = 45)	Study group (n = 45)	Statistical value	P
Age ( $\bar{X}$ ± s, year)		40.24 ± 10.26	40.28 ± 10.41	*t* = 0.018	0.985
Duration of ICU stay ( $\bar{X}$ ± s, day)		7.26 ± 2.52	7.24 ± 2.59	*t* = 0.037	0.971
Sex [n (%)]	Male	21 (46.67)	23 (53.11)	χ^2^ = 0.178	0.673
	Female	24 (53.33)	22 (48.89)
APACHE II ( $\bar{X}$ ± s, point)		17.09 ± 1.52	17.06 ± 1.53	*t* = 0.093	0.926

### Ward Management Quality Score

The differences in the ward management quality scores of all items were not significant between the study group and the control group before management (P > 0.05). The study group had higher ward management quality scores of all items than the control group after management (P < 0.05) (Table 2).

**Table 2:** Comparison of ward management quality scores ( $\bar{X}$ ± s, point) – Wenzhou, Zhejiang Province, China, 2021–2023.

Group	n	Hygienic environment		Management of items		Treatment and nursing level of medical staff		Implementation of safety measures	
		Before management	After management	Before management	After management	Before management	After management	Before management	After management
Control	45	13.72 ± 1.52	17.11 ± 2.22^a^	12.26 ± 1.35	16.32 ± 2.30^a^	13.25 ± 1.25	18.84 ± 2.05^a^	11.56 ± 1.24	18.30 ± 2.50^a^
Study	45	13.74 ± 1.55	18.50 ± 2.25^a^	12.29 ± 1.36	17.95 ± 2.28^a^	13.28 ± 1.24	20.20 ± 2.08^a^	11.58 ± 1.26	20.10 ± 2.55^a^
*T*		0.062	2.950	0.077	3.376	0.114	3.124	0.076	3.371
P		0.951	0.004	0.939	0.001	0.909	0.002	0.940	0.001

^a^P < 0.05 *vs*. the same group before management.

### Nursing Quality Scores

The nursing quality scores of all items had no significant differences between the study group and the control group before management (P > 0.05). In the study group, the nursing quality scores of all items were significantly higher than in the control group after management (P < 0.05) (Table 3).

**Table 3:** Nursing quality scores ( $\bar{X}$ ± s, point) – Wenzhou, Zhejiang Province, China, 2021–2023.

Group	n	Service attitude		Theoretical assessment		Practical assessment		Nursing satisfaction	
		Before management	After management	Before management	After management	Before management	After management	Before management	After management
Control	45	13.62 ± 2.30	18.40 ± 2.28^a^	12.28 ± 1.31	18.40 ± 2.37^a^	13.26 ± 1.22	18.89 ± 1.80^a^	11.58 ± 1.21	18.50 ± 2.10^a^
Study	45	13.60 ± 2.31	20.00 ± 2.30^a^	12.26 ± 1.33	20.05 ± 2.35^a^	13.24 ± 1.20	20.20 ± 1.90^a^	11.55 ± 1.22	20.00 ± 2.15^a^
*t*		0.041	3.314	0.072	3.316	0.078	3.358	0.117	3.348
P		0.967	0.001	0.943	0.001	0.938	0.001	0.907	0.001

^a^P < 0.05 *vs*. the same group before management.

### Level of Comfort

The GCQ scores had no significant differences between the study group and the control group before management (P > 0.05). In the study group, the GCQ scores were significantly higher than in the control group after management (P < 0.05) (Table 4).

**Table 4:** Level of comfort ( $\bar{X}$ ± s, point) – Wenzhou, Zhejiang Province, China, 2021–2023.

Group	n	Before management	After management	*t*	P
Control	45	70.86 ± 4.25	80.35 ± 5.84	8.814	0.000
Study	45	70.49 ± 4.99	98.84 ± 6.52	23.163	0.000
*t*		0.379	14.170		
P		0.706	0.000		

### Severity of Disease

The APACHE II scores had no significant differences between the study group and the control group before management (P > 0.05). The APACHE II scores significantly declined in the study group compared with those in the control group after management (P < 0.05) (Table 5).

**Table 5:** APACHE II scores ( $\bar{X}$ ± s, point) – Wenzhou, Zhejiang Province, China, 2021–2023.

Group	n	Before management	After management	*t*	P
Control	45	17.09 ± 1.52	13.35 ± 0.84	14.447	0.000
Study	45	17.06 ± 1.53	11.84 ± 0.52	21.670	0.000
*t*		0.093	10.253		
P		0.926	0.000		

### Incidence of Adverse Events

The incidence of adverse reactions during management in the study group was slightly lower than in the control group, but the difference was not significant (P > 0.05) (Table 6).

**Table 6:** Incidence of adverse events [n (%)] – Wenzhou, Zhejiang Province, China, 2021–2023.

Group	n	Delayed or incorrect implementation of medical orders	Cross-infection	Unplanned extubation	Insufficient equipment power	Insufficient consumables	Total
Control	45	1 (2.22)	1 (2.22)	1 (2.22)	1 (2.22)	1 (2.22)	5 (11.11)
Study	45	0 (0.00)	0 (0.00)	1 (2.22)	1 (2.22)	0 (0.00)	2 (4.44)
χ^2^							0.620
P							0.431

## DISCUSSION

Compared to other departments, ICU is characterized by its high unpredictability, moderate workload, and fast-paced environment. These factors contribute to higher nursing risks and a greater risk of nursing adverse events. With a robust management system, nursing risks can be promptly addressed or mitigated by appropriate management measures. This approach helps reduce adverse events and prevents potential medical disputes^(14,15)^. At the time this study was conducted, routine programmed management was mostly used in the ICU, which has achieved some effects. However, the overall management effect still needs improvement, indicating the need for more efficient management measures^(16,17)^.

In this study, the ward management quality scores and nursing quality scores of all items were significantly higher in the study group than in the control group, suggesting that combining the 9S management theory with hierarchical management model can effectively enhance the ward management quality and nursing quality in the ICU settings. The reasons are as follows. Firstly, using the 9S management theory, the functional zoning of the ward is rationally planned. Medical items, equipment and consumables are organized through SEIRI and SEITON, improving environmental cleanliness in the ward. SEISO and SEIKETSU help eliminate hygienic dead zones and ensure strict implementation of disinfection and isolation protocols, maintaining a clean and hygienic ward environment. These measures create a clean working and medical care environment for both doctors and patients^(18,19)^. Secondly, by applying the 9S management theory, accounts are established to effectively save on consumables, preventing the expiration of disposable items and ensuring adequate quantities when needed. Moreover, instruments and equipment are regularly maintained by designated personnel. Medical staff are required to dispose of garbage and medical waste according to strict protocols and regulate inspections of the ward’s environment and facilities are conducted to eliminate potential safety hazards. These measures significantly improve the efficiency of item management and enhance the safety management level in the ward^(20)^.

Thirdly, the 9S management theory involves several measures, such as exchange of learning, theoretical and practical assessments, and also special learning activities of medical ethics and technology, in order to enhance the professional level of medical and nursing staff. High-quality hierarchical training activities effectively improve the nursing service level and service attitude of medical and nursing staff. By conducting questionnaire surveys with patients transferred out of the ward, current nursing service deficiencies can be accurately identified and addressed. These measures help enhance the treatment and nursing quality of medical and nursing staff, and also improve patient satisfaction^(21)^.

Moreover, in this study, the study group had higher GCQ scores and lower APACHE II scores than those of the control group after management, suggesting that combing the 9S management theory with hierarchical management model can positively impact patient comfort and reduce the severity of disease in ICU management. The reasons are as follows: 1 - by enhancing the professional level of medical and nursing staff, providing high-quality nursing services, and assessing nursing satisfaction in the 9S management theory, patient comfort can be effectively improved. This, in turn, helps to improve patient conditions and reduce the severity of disease^(22)^; 2 - patients are categorized into different grades by hierarchical management based on the severity of their conditions. Corresponding nursing measures are then taken in a targeted, effective and scientific manner, which markedly improves patient comfort and reduces the severity of disease^(23)^. In addition, the incidence rate of adverse reactions during management in the study group was slightly lower than that of the control group, but the difference was not statistically significant. This suggests that the incidence of adverse events is comparable between 9S management theory with the hierarchical management model and routine management in ICU settings.

Nevertheless, the small sample size and short intervention period may have influenced the results of this study. Therefore, large-scale randomized controlled studies are needed in the future to provide an accurate and detailed theoretical basis for evaluating adverse clinical events.

## CONCLUSION

In conclusion, combining the 9S management theory with the hierarchical management model can effectively improve ward management quality and nursing quality in ICU settings. This approach has significant benefits for enhancing patient comfort and reducing the severity of disease. This combined nursing plan demonstrates prominent advantages.
